# GAIT ANALYSIS OF ELDERLY PEOPLE IN ANGOLA: COMPARISON BETWEEN ESTABLISHED ROUTE AND VIRTUAL REALITY AND TIME UP AND GO

**DOI:** 10.1093/geroni/igad104.2458

**Published:** 2023-12-21

**Authors:** Rita Barbara, Natália Luanda

**Affiliations:** Universidade de Aveiro, Sao Paulo, Sao Paulo, Brazil; Upra, Luanda, Luanda, Angola

## Abstract

**Background:**

Aging limits physical and mental abilities, particularly gait, compromising quality of life and increasing the risk of falls, which is a public health challenge in Angola. Technologies, such as virtual reality, may offer a promising alternative for treatment and prevention of these limitations. Objective: This study aimed to analyze the gait of elderly individuals in Angola by comparing gait on an established course and in a virtual environment, using motion analysis software and SPSS for statistical analysis with a p>0.05.

**Methods:**

Seven elderly individuals, aged 60-80, participated in the study. Gait was evaluated using the Timed Up and Go (TUG) test, an established course, and gait in a virtual environment.

**Results:**

Statistically significant differences were found between gait on the established course and in virtual reality, with elderly individuals showing slower and more variable gait in the virtual environment. There were also significant correlations between gait and TUG, highlighting the importance of motor coordination and balance in elderly gait.

**Conclusion:**

Gait analysis in elderly individuals can be performed precisely and stably through virtual reality technology. The results of this study suggest that virtual reality may be an interesting alternative for the treatment and prevention of falls in the elderly. The findings also emphasize the importance of motor coordination and balance in gait and the need for further research on the use of virtual reality technology in this population.

